# Development of a Modular Assay for Detailed Immunophenotyping of Peripheral Human Whole Blood Samples by Multicolor Flow Cytometry

**DOI:** 10.3390/ijms17081316

**Published:** 2016-08-11

**Authors:** Paul F. Rühle, Rainer Fietkau, Udo S. Gaipl, Benjamin Frey

**Affiliations:** Department of Radiation Oncology, Universitätsklinikum Erlangen, Friedrich-Alexander-Universität Erlangen-Nürnberg, Erlangen 91054, Germany; paul.ruehle@uk-erlangen.de (P.F.R.); rainer.fietkau@uk-erlangen.de (R.F.); benjamin.frey@uk-erlangen.de (B.F.)

**Keywords:** immune monitoring, multicolor flow cytometry, immunophenotyping, liquid biopsy, whole blood, innate immune system, adaptive immune system

## Abstract

The monitoring of immune cells gained great significance in prognosis and prediction of therapy responses. For analyzing blood samples, the multicolor flow cytometry has become the method of choice as it combines high specificity on single cell level with multiple parameters and high throughput. Here, we present a modular assay for the detailed immunophenotyping of blood (DIoB) that was optimized for an easy and direct application in whole blood samples. The DIoB assay characterizes 34 immune cell subsets that circulate the peripheral blood including all major immune cells such as T cells, B cells, natural killer (NK) cells, monocytes, dendritic cells (DCs), neutrophils, eosinophils, and basophils. In addition, it evaluates their functional state and a few non-leukocytes that also have been associated with the outcome of cancer therapy. This DIoB assay allows a longitudinal and close-meshed monitoring of a detailed immune status in patients requiring only 2.0 mL of peripheral blood and it is not restricted to peripheral blood mononuclear cells. It is currently applied for the immune monitoring of patients with glioblastoma multiforme (IMMO-GLIO-01 trial, NCT02022384), pancreatic cancer (CONKO-007 trial, NCT01827553), and head and neck cancer (DIREKHT trial, NCT02528955) and might pave the way for immune biomarker identification for prediction and prognosis of therapy outcome.

## 1. Introduction

In the last decades, immunotherapy (IT) has become a prominent part in multimodal cancer therapy complementing the classical treatments of surgery, chemotherapy (CT) and radiotherapy (RT). It has successfully been established for certain cancers, but unfortunately not all cancer therapies benefit from its promising potential. Furthermore, challenges exist in finding optimal combinations and suited time points for its inclusion. Here, the knowledge of the immune status during therapy is becoming increasingly important particularly in the prediction and prognosis of therapy responses in multimodal cancer treatments [1].

It has become clear that classical tumor therapies such as RT and CT do not only destroy tumor cells, but also modulate their phenotype and, especially in the combination with further IT, can initiate systemic immune-mediated anti-tumor responses [2]. Once the relationships between tumor stage, therapy and immune status have been identified, prognostic and predictive markers might be derived [3,4,5]. Thereby, one big challenge is to monitor the immune status in a close-meshed manner to identify optimal time points for integration of IT into existing RT/CT protocols [6]. Apparently, the immune monitoring would ideally be performed in the affected tissues. However, these are not always accessible or a repetitive extraction is prohibited. Thus, liquid biopsies such as whole blood are mandatory in addition to solid biopsies that only give hints on the immune status at restricted time points of the disease due to limited availability.

Indeed, the peripheral blood is of great significance for a close-meshed immune monitoring because it is relatively easy to obtain and still carries a high informative value as the immune cells pass it to reach their target tissues. Thus, immune modulations in the distant tumor microenvironment might also affect the immune status in the peripheral blood allowing the recognition of therapy responses [7]. Consequently, the immune monitoring of blood is ideal for the analysis of cancer progression and therapeutic outcomes [8] complementing standard analyses performed with solid biopsies [9]. Here, the multicolor flow cytometry can easily make its way into clinical routine, especially, when blood is the biomaterial. The possibility of measuring multiple parameters at once on a single-cell level combined with a high throughput makes flow cytometry to one of the most powerful technologies for determining cell subsets in a mixed suspension [10].

Over the last years, several groups have developed multicolor flow cytometry-based assays that are suitable for an immune monitoring of patients. These assays widely differ in their level of detail ranging from one cell type [11,12,13] over lymphocytes [14] or myeloid cells [15] to a comprehensive immune status [16,17,18] from which, however, often the granulocytes (neutrophils, eosinophils and basophils) were omitted [17,18]. Recently, the focus was furthermore set on the establishment of harmonized assays that are suited for an application in multi-centric analyses [18,19,20]. These assays often include the pre-analytic isolation of peripheral blood mononuclear cells (PBMC) to enhance the sample durability which allows sample storage and long-term shipments. However, as this procedure is time consuming and omits certain cell types, it also carries some disadvantages.

We present here a multicolor flow cytometry-based assay that examines the detailed immune status covering 34 different immune cell subsets and three non-immune cell subsets in only 2 mL of human peripheral blood. It was optimized for a direct staining of whole blood samples which on the one hand allows the detection of all circulating immune cells and on the other hand reduces the required preparation steps. Thus, in addition to minimizing effort and variations in sample preparation, the direct staining procedure also is time-saving, a further prerequisite for an easy clinical application, involving less than 20 min hands-on time. The assay was designed to allow a detailed immunophenotyping of blood (DIoB) identifying almost all circulating immune cells. These cover all major immune cell types such as T cells, B cells, natural killer (NK) cells, dendritic cells (DCs), monocytes, neutrophils, eosinophils, and basophils, as well as circulating stem cells, progenitor cell and endothelial cells which already have been connected to certain cancer therapy responses. Moreover, the functional state is analyzed by the additional staining for activation markers. The DIoB assay was designed in a modular principle comprising 12 panels which each is dedicated to determine one specific type of cell and its subsets. In total, it is suited for the monitoring of the detailed immune status of patients in short intervals paving the way for optimization or even individualization of multimodal cancer therapies.

## 2. Results

### 2.1. Examining 37 Cell Subsets

**Figure 1:**
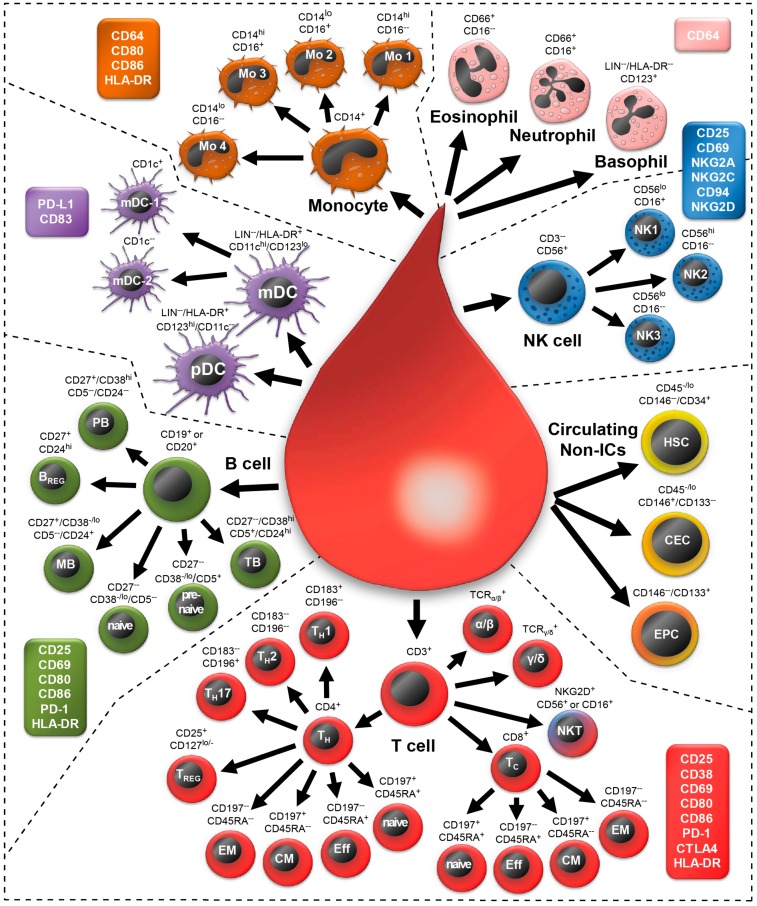
Schematic overview of the 34 immune cell and 3 non-immune cell subsets that can be identified by the here presented detailed immunophenotyping of blood (DIoB) assay. Whole blood samples were stained with specific antibody mixes in 11 different panels. Thus, all major circulating leukocytes such as T cells (**red**), B cells (**green**), dendritic cells (DC; **violet**: myeloid DC, plasmacytoid DC), monocytes (**brown**), granulocytes (**pink**: eosinophils, neutrophils, basophils), and natural killer (NK) cells (**blue**) were detected. Additionally, circulating non-immune cells (**yellow**/**orange**) such as hematopoietic stem cells (HSC), circulating endothelial cells (CEC) and endothelial progenitor cells (EPC) were monitored. These main cell types are depicted as bigger cells and all subsets, which were differentiated out of them, are depicted as smaller cells. For all of them, the markers that are necessary for their identification are indicated. This overview is completed by the enumeration of 27 activation markers which were assigned to corresponding cell types (colored boxes). The arrows represent the gating strategy in a simplified manner.

The DIoB multicolor flow cytometry assay allows the identification of 34 well defined immune cell subsets in human peripheral whole blood samples (Figure 1). These encompass all major immune cell types which are differentiated into 34 different subsets. Additionally, three non-immune cells which have been associated with disease progression are evaluated. The DIoB assay was designed in a modular system comprising 12 different panels (Table 1). Thereof, 11 panels (P01–P11) are each dedicated to one cell type. In contrast, the 12th panel determines the absolute cell count of the identified subsets. Additionally, 27 activation markers were included for determination of the activation state of these cells. The gating strategy for identification of these cells and its activation states is outlined in the following results sections including their phenotypical descriptions in the literature. An overview of definitions for each cell is provided in Table 2.

### 2.2. Morphology of All Leukocytes

The identification of all subsets was performed by the analysis of surface antigens. However, the first step in all panels was the definition of a few identical gates, which were based on the morphologic properties of the cells, to discriminate the unwanted events from leukocytes and thus creating a consistent basis for the subsequent surface marker investigations.

**Figure 2:**
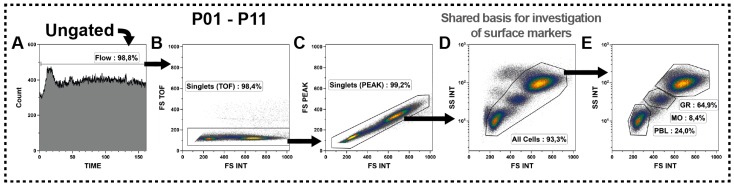
Definition of the All Cells-gate as crucial basis for the subset identifications by surface markers. (**A**) Acquisition characteristics were evaluated and irregularities were excluded by definition of Flow-gates for each panel; (**B**,**C**) Then, doublets were excluded by cross-checking the forward scatter (FSC) signal for its integral (INT) versus time of flight (TOF) and peak (PEAK) characteristics; (**D**) Finally, the All Cells-gate was defined based on its scatter characteristics. Hereby, the events that shifted to a lower FSC signal were considered as dead or dying cells and removed together with the debris from that definition; (**E**) The All Cells-gate could be subdivided on its scatter characteristics into granulocytes (GR), monocytes (MO) and lymphoid cells (PBL); and (**A**–**E**) Black arrows represent the gating strategy and the depicted percentages depend on the respective input gate.

First, the Flow-gates were defined analyzing the event count against the time parameter (Figure 2A) to check for irregularities during acquisition and to discriminate these. Then, cell doublets were excluded by the integral of the forward scatter (FSC) signal (area of signal) versus the FSC time of flight (width of signal; Figure 2B) followed by FSC integral vs. FSC peak (height of signal; Figure 2C). Subsequently, the All Cells-gate was defined, representing the circulating leukocytes and non-leukocytes, based on its FSC (size) and side scatter (SSC: complexity) parameters (Figure 2D). We only considered cells that were in good shape and excluded all events that had a lowered FSC signal (Figure 2D). This All Cells gate might further be distinguished into lymphoid cells (PBL, small and less complex), monocytes (Mo, more complex) and granulocytes (Gr, biggest and most complex) as shown in Figure 2E. However, the subsequent identification of cell subsets should be performed on basis of all leukocytes (All Cells-gate).

These few morphology gates were defined in the same manner for all panels, except P12, to ensure the examination of the same set of events in all panels. Eventually, similar gates were defined for P12 by adjusting for its scatter characteristics, meaning higher FSC properties because the sample had not been centrifuged (described below in Section 2.11).

Intentionally, we did not include a dead cell marker as dead cells were not a big issue in fresh whole blood samples and were always below 1% as confirmed by preceding analyses (not shown). Besides, we observed that almost all propidium iodide (PI) positive cells were located within the cells that shifted to a lower FSC, which were excluded anyway. Nevertheless, if some users wish to include a dead cell marker, PI would be ideal since the corresponding fluorescence channel was left blank in all panels.

### 2.3. T Cell Subsets

The T cell subsets are the most intensively studied ones and with about 20%–30% also the second most common in the peripheral blood. Thus, until today a plethora of different subsets were identified and characterized. Likewise, our assay identified the most subsets within the T cells which were determined in P01, P02 and P03 (Table 1: red rows). Additionally, their activation state was determined in P05 together with B cells (Table 1: red/green row).

**Figure 3:**
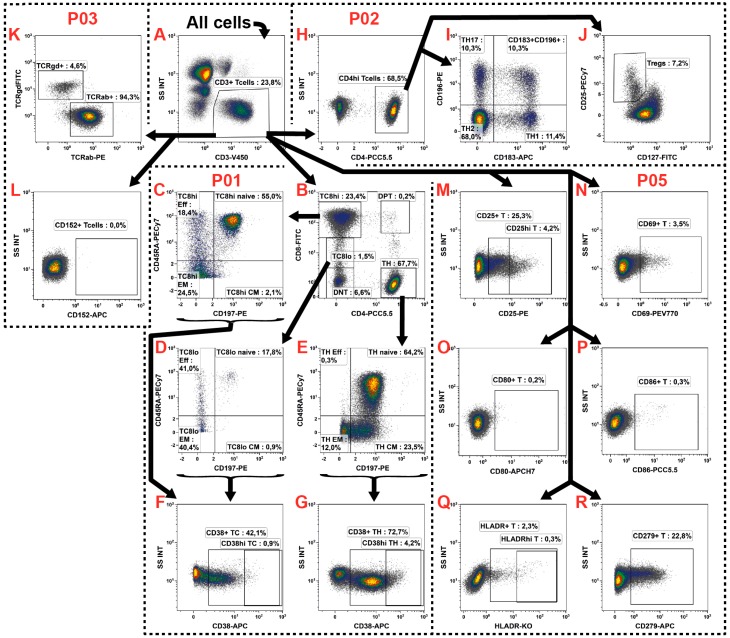
Gating strategy for the identification of fourteen T cell subsets and the determination of their activation state. (**A**) The T cells were identified by their CD3 expression defining the same gates for the panels P01, P02, P03 and P05; (**B**–**G**) The P01 was used for the identification of T_C_ and T_H_ subsets; (**B**) Thus, first the T_H_ and T_C_ were identified by their differential CD4 and CD8 expression, whereby the T_C_ were differentiated into CD8^hi^ and CD8^lo^ populations. Additionally, the double negative (DNT) and double positive (DPT) T cells were recorded; (**C**–**E**) The T_H_, T_C_8^hi^ and T_C_8^lo^ were further distinguished into naïve, effector (Eff), effector memory (EM), and central memory (CM) subsets by their CD197 and CD45RA expression; (**F**,**G**) In order to determine the activation state of these subsets, the CD38 expression was examined; (**H**,**I**) In P02, the T_H_ were differentiated into T_H_1, T_H_2, and T_H_17 by their CD186 and CD196 co-expressions; (**J**) In addition, the T_REG_ were identified by their CD25^hi^/CD127^-/lo^ phenotype; (**K**,**L**) The P03 was introduced for identification of the general TCR expression as well as the examination of the regulation of the immunosuppressive CTLA-4; (**M**–**R**) Finally, the P05 investigated the activation state of T cells in general, examining the expression of CD25, CD69, CD80, CD86, and HLA-DR, as well as that of the CD279; and (**A**–**R**) Black arrows represent the gating strategy and the depicted percentages depend on the respective input gate.

First, in all four panels the same CD3^+^ gate was defined (Figure 3A) followed by individual sub-gating. In P01 the CD4^+^ T helper cells (T_H_) and CD8^+^ cytotoxic T cells (T_C_) were distinguished whereby T cells expressing both antigens were excluded from these definitions (Figure 3B). For T_H_ definition, only CD4^hi^ cells were considered. In contrast, we included both the CD8^hi^ and CD8^lo^ cells into the definition of T_C_ as CD8 might be down-regulated in certain subsets [21]. These cells were analyzed individually (T8^lo^ and T8^hi^) and subsequently merged into all T_C_ by the definition of a Boolean gate (see Table 3). The remaining T cells (5%–10%) differed from the classical view of circulating T cells and thus were excluded from continuing examinations, but were still recorded as double negative (DNT: CD4^−^/CD8^−^), double positive (DPT: CD4^hi^/CD8^hi^), CD4^lo^/CD8^−^, CD4^hi^/CD8^lo^ or CD8^hi^/CD4^lo^ T cells (Figure 3B: for the sake of clarity the last three gates are not shown). We never observed a subset with jointly low expression of both markers (CD4^lo^/CD8^lo^). This analysis was continued by individual subdivisions of T_H_ and T_C_ by their CD45RA and CD197 (CCR7) expression into naïve (CD45RA^+^/CD197^+^), effector (T_EFF_, CD45RA^+^/CD197^−^), effector memory (T_EM_, CD45RA^−^/CD197^−^) and central memory (T_CM_, CD45RA^−^/CD197^+^) T cells (Figure 3C–E), as previously described by [22,23].

In P02 the T_H_ were further distinguished into the T_H_1 (CD183^+^/CD196^−^), T_H_2 (CD183^−^/CD196^−^) and T_H_17 (CD183^−^/CD196^+^) subsets (Figure 3H,I), as described by [24,25,26]. A fourth heterogeneous T_H_ population (Figure 3I: CD183^+^/CD196^+^) was observed which was described to comprise T_H_1 and cells producing both IFNγ and IL-17 [24]. Furthermore, the regulatory T cells (T_REG_) were identified by their CD25^hi^/CD127^−/lo^ phenotype [27,28] as shown in Figure 3J. The panel P03 was included to explore the TCR expression and to thereby distinguish the T cells into TCRα/β^+^ and TCRγ/δ^+^ T cells [29] (Figure 3K).

In addition to the subset identification, the T cells were investigated for their activation state. This, on the one hand, was explored for the different T_H_ and T_C_ subsets in P01 by analyzing the expression of the typical T cell activation marker CD38 [30] (Figure 3E–G). On the other hand, common lymphocyte activation markers such as CD25 (IL2 receptor), CD69 (very early activation antigen), CD80 (B7-1) and CD86 (B7-2) were determined in P05 for the general T cell population without distinguishing any subsets (Figure 3M–P). In addition, the expression of HLA-DR was monitored (Figure 3Q) which might be expressed upon activation [31]. Such HLA-DR^+^ T cells are able to present auto antigens to other T cells and thus directly suppress them [32]. Moreover, the immune checkpoint protein CD279 (PD1: Programmed Cell Death Protein 1) was monitored in P05 (Figure 3R). It plays an important role in T cell balance and immune tolerance and has already been described to be regulated in certain cancers (reviewed in [33]) and is also a target for IT [34]. In parallel, its ligands CD274 (PD-L1), CD80, and CD86 that are expressed by other immune cells such as DCs and monocytes were monitored in the respective panels (described below). In parallel, also the expression of the immunosuppressive CD152 (CTLA-4: cytotoxic T-lymphocyte-associated protein 4) was analyzed on T cells in P03 (Figure 3L). The CD152 is of clinical interest as it is, like CD279 (PD1), a prominent suppressor of T cell activation and upregulated following T cell activation to prevent an excessive immune reaction. It moreover also represents a target for IT (reviewed in [2]).

### 2.4. B Cell Subsets

The B cells belong with about 5% of all leukocytes to the less common peripheral cells and unfortunately also their subset distinction is not always uniform. Even though their role in the circulation is not completely understood, their number was described to be altered in numerous diseases and cancers and therefore should be monitored. We distinguished six well-described subsets in P04 (Table 1: green row) and examined the activation state of B cells in P05 (Table 1: green/red row).

**Figure 4:**
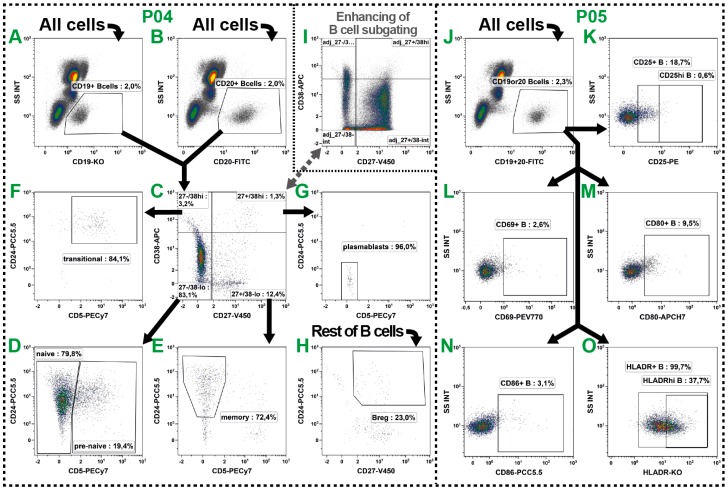
Gating strategy for the identification of six B cell subsets (P05: **A**–**I**) and determination of their activation state (P06: **K**–**P**). (**A**,**B**) For the definition of B cells, the expression of CD19 (**A**) and CD20 (**B**) was initially individually investigated, and then merged by the definition of a Boolean gate for subsequent analyses; (**C**–**H**) Following, the subsets were characterized in a two-step process by their expression of CD27, CD38, CD5 and CD24; (**C**) First, a CD27 vs. CD38 quadrant was defined; (**D**–**G**) Then, these four gates were investigated for their CD5 vs. CD24 co-expression allowing the definition of pre-naïve (**D**), naïve (**D**), memory (**E**) and transitional B cells (**F**) as well as plasmablasts (**G**); (**H**) All B cells not belonging to one of these subsets were defined as Rest of B cells by a Boolean gate and analyzed for their CD27^+^/CD24^hi^ phenotype to identify the B_REGs_; (**I**) As B cells are sometimes sparsely distributed especially in cancer patients, the gate settings might be aligned comparing the expression patterns on PBL level; (**J**–**O**) In addition, common activation markers were examined together with T cells in P05. Therefore, CD19 and CD20 were combined into one fluorescence channel (**J**). Then, the expression of CD25 (**K**), CD69 (**L**), CD80 (**M**), CD86 (**N**), and HLA-DR (**O**) was analyzed; and (**A**–**O**) Black arrows represent the gating strategy and the depicted percentages depend on the respective input gate.

To identify B cells the pan marker CD19 is widely used, but has the disadvantage of low expression and down-regulation in certain diseases and cancer [35]. Consequently, we additionally included CD20 as another common B cell marker which is expressed at high levels on most B cells. Both markers were separately analyzed in P04 (Figure 4A,B), but combined prior to the subset examinations by defining a Boolean gate (CD19 or 20 B cells; see Table 3). Consequently, the potential regulation of one of these markers could be estimated in the course of different blood withdrawals.

Then, in a two-step gating process the B cells were further divided into six subsets by their differential expression of CD27, CD38, CD5 and CD24 (summarized in [36,37]) (Figure 4C–G). Thus, we identified pre-naïve [38] (Figure 4D: CD27^−^/CD38^−/lo^/CD5^+^), naïve [38,39,40] (Figure 4D: CD27^−^/CD38^−/lo^/CD5^−^), memory [39,40,41] (Figure 4E: CD27^+^/CD38^−/lo^/CD5^−^/CD24^+^) and transitional B cells [42,43] (Figure 4F: CD27^−^/CD38^hi^/CD5^+^/CD24^+^), as well as plasmablasts [44] (Figure 4G: CD27^+^/CD38^hi^/CD5^−^/CD24^−^). In addition, we identified the rare immunosuppressive B10 regulatory B cells (B_REG_) which do not express a unique set of surface markers but are CD27^+^/CD24^hi^ [45]. Thus, we gated on all CD27^+^/CD24^hi^ B cells after the exclusion of the other five subsets. Therefore, a Boolean gate was defined and labeled as Rest of B cells which covered all B cells that were not located in the pre-naïve, naïve, memory, transitional or plasmablast gates (see Table 3). These cells were then investigated for their CD27 and CD24 expression (Figure 4H).

The activation state of B cell subsets was examined in P05. For this, both pan markers (CD19 and CD20) were combined into the same fluorescence channel (Figure 4J) resulting in the saving of one channel. Subsequently, the expression of CD25, CD69, CD80, CD86 and HLA-DR was examined on B cell level (Figure 4K–O), as described above for the T cells.

### 2.5. Natural Killer Cells

The distribution of the innate NK cells is with 3%–5% similar to that of B cells. In general, NK cells are identified by their CD56 expression while lacking the common T cell marker CD3 [46] and are then distinguished into different subsets by their CD16 (FCγRIIIA) co-expression [47]. Despite, also NK cells lacking of CD56 expression exist, in particular in the periphery of patients with chronic viral infections [48]. For their evaluation as well as further distinction of NK cells in up to five subsets a comparison of the co-expression of CD94 [49] and/or CD57 [50] could be taken into account (discussed in [51]).

Investigating the CD16 molecule on NK cells one should pay attention in choosing an antibody clone which specifically detects its isoform A. Although the antibody clone 3G8 is widely used, we included the B73.1 clone as we obtained better results in whole blood staining. In addition to NK cells also monocytes express the isoform A of the FcγRIII, but neutrophils express FcγRIIIB (summarized in [52]). Both isoforms are up to 95% identical; however, the B73.1 clone specifically binds to the FcγRIIIA and the 3G8 clone binds both isotypes [53]. Consequently, using the B73.1 clone results in a stable staining of CD16^+^ NK cells and monocytes even in neutrophil-rich patient blood samples. However, this advantage might be tarnished as there is a rare mutation in the CD16a molecule leading to loss of the binding epitope for the B73.1 antibody. This rare mutation is linked to primary NK cell immunodeficiency and repeated infections by herpes viruses [54]. Though, it is advised to apply both clones in persons with suspicion of such a CD16a mutation or when analyzing study populations of primary NK cell immunodeficiencies.

**Figure 5:**
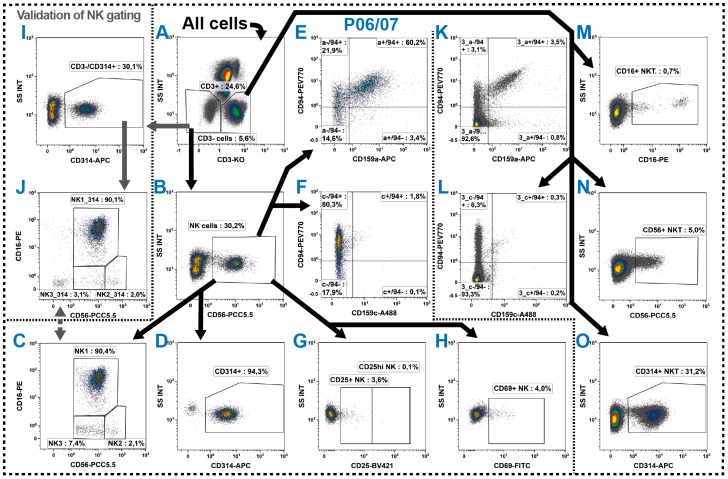
Gating strategy for the identification of three NK cell subsets (**A**–**C**) and their functional states (**D**–**H**), as well as several NKT cell subsets (**K**–**O**). (**A**–**C**) The NK cells were determined in P06 and P07 as CD3^−^/CD56^+^ cells and subsequently distinguished into three subsets by their CD56 and CD16 co-expression into NK1, NK2 and NK3; (**D**–**F**) Their cytotoxic activity was determined examining the expression of CD314, the suppressing CD159a and the activating CD159c, whereby the latter two were analyzed in co-expression to CD94; (**G**,**H**) In addition, their activation state was determined by examining the expression of CD25 and CD69; (**I**,**J**) As CD56 is generally low expressed, the NK cell identification was cross-checked by the CD314 expression on CD3^−^ cells and also the three subsets were validated; (**J,K**) In parallel, NKT cells were determined as all CD3^+^ cells which simultaneously expressed one of the typical NK cell markers CD16, CD56, CD94, CD159a, CD159c or CD314; and (**A**–**O**) Black arrows represent the gating strategy and the depicted percentages depend on the respective input gate.

In the DIoB assay, we focused on the CD56^+^ NK cells (Figure 5A,B) and distinguished them into three different subtypes (Figure 5C) in both NK cell panels P06 and P07 (Table 1: blue rows) which only differ in the examination of functional markers. The CD56^lo^/CD16^+^ cells (here termed *NK1*) accounted with nearly 90% by far for the main subset. These NK1 cells were described as predominantly cytotoxic [47,55,56]. In contrast, the CD56^hi^/CD16^−^ cells (here termed NK2) were described as having a rather supporting role by predominantly secreting cytokines [47,55,57]. In addition, we summarized the CD56^lo^/CD16^−^ NK cells as NK3 subset. This subset has functionally not been described yet, but was proposed to be in-between developmental states [58]. All these gates were linked between P06 and P07.

Most NK cells express CD314 (NKG2D: natural-killer group 2 member D) [59,60,61] (Figure 5D). Thus, this marker can also be useful for the cross-checking of the NK cell identification (Figure 5I,J) as the low expression of CD56 sometimes complicates the NK cell identification. However, it should be noted that the CD314 expression might be regulated under certain conditions [62,63,64] making it inappropriate for a direct NK cell identification.

In order to determine the activation state, the common lymphocyte activation markers CD25 (Figure 5G) and CD69 were analyzed (Figure 5H) in P06 as suggested by [65,66,67]. Furthermore, we characterized the NK cells by their expression of CD159a (NKG2A), CD159c (NKG2C) and CD94. The CD159a and CD159c form heterodimers with CD94 and function as inhibitory (CD94/NKG2A) or activating (CD94/NKG2C) receptor complex (summarized in [68]) and consequently were analyzed in co-expression (Figure 5E,F). For the determination of those molecules on the different NK cell subsets, Boolean gates were defined accordingly (not shown).

### 2.6. NKT Cells

The NKT cells are a not-uniformly defined type of cells expressing markers from both T and NK cells. Consequently, they are defined as CD3 positive cells that simultaneously express CD56, CD314 or CD16 and have been described in several studies such as [69,70,71,72,73,74]. Thus, we investigated the expression of CD56, CD16, CD314, CD159a, CD159c and CD94 on all CD3^+^ cells in parallel to the expression on NK cells in the panels P06 and P07 (Figure 5K–O). Then, all cells belonging to a least one of these gates were termed NKT cells. For determination of NKT cell prevalence, Boolean gates were defined to prevent the repeated counting of cells which belonged to more than one of the NKT gates (see Table 3).

### 2.7. Monocytes

The monocytes constitute for 7%–10% of all leukocytes and have typical SSC properties in-between that of the SSC^hi^ granulocytes and that of the SSC^lo^ lymphoid cells. However, with CD14 they also express a very characteristic pan marker. Their subset definition is not always uniform, but it is generally accepted to divide them by their CD16 (FCγRIIIA) co-expression into two, three or even four subsets. These subsets could then further be subdivided by their co-expression of other markers such as HLA-DR. The nomenclature of monocyte subsets is well-summarized in [75]. As already mentioned for the NK cell subsets, one should concern choosing an antibody-clone which binds the isoform A of the CD16-molecule. Moreover, monocytes generally have a high unspecific binding capacity and therefore all antibodies used for their characterization have to be checked very carefully. Furthermore, it already has been described that monocytes also bind certain fluorochrome dimers such as PE-Cy5 [76], PE-Cy7 and APC-Cy7 [77] and we observed the same to be true for the PE-Vio770. Consequently, these fluorochromes should not be used for monocyte evaluation.

**Figure 6:**
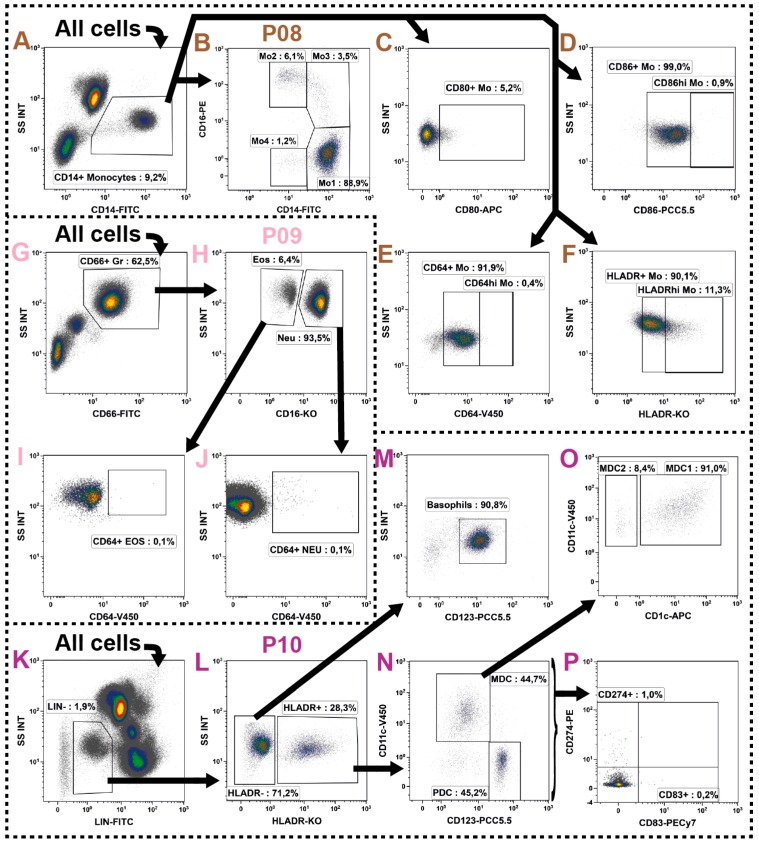
Gating strategy for identification of myeloid cells such as monocytes (P08: **A**–**F**), neutrophils and eosinophils (P09: **G**–**J**), as well as basophils and dendritic cells (P10: **K**–**O**). (**A**,**B**) The monocytes were identified in P08 by their CD14 expression and subsequently distinguished into four subsets by their CD16 co-expression; (**C**–**F**) For determination of their activation state, the expression of CD80, CD86, CD64 and HLA-DR was analyzed; (**G**,**H**) In P09 the neutrophils (Neu) and eosinophils (Eos) were identified by their shared expression of CD66 and high SSC characteristics, but differential CD16 expression; (**I**,**J**) Then, both cell populations were examined for their expression of CD64 as an activation marker. Here, attention should be paid to the high auto fluorescence characteristics of eosinophils; (**K**) In P10, the DCs and basophils were examined following the exclusion of most other cells by their lineage markers (LIN: CD3, CD14, CD16, CD19, CD20, CD56); (**L**,**M**) Then, the basophils were identified by their lack of HLA-DR while expressing CD123; (**L**,**N**) In contrast, the DCs express HLA-DR and can be distinguished by their differential expression of CD11c and CD123 into pDC and mDC; (**O**) The mDC were further divided into mDC-1 and mDC-2 by their differential expression of CD1c; (**P**) Finally, the maturation marker CD83 and the PD1 ligand CD274 (PD-L1) were determined on all DCs; and (**A**–**O**) Black arrows represent the gating strategy and the depicted percentages depend on the respective input gate.

The DIoB assay identifies the monocytes in P08 (Table 1: brown row) by their CD14 expression (Figure 6A) and subdivides them by their CD16 co-expression into four subsets (Figure 6B). The CD14^hi^/CD16^−^ monocytes obviously accounted with 80%–90% for the main subset. This subset is often referred to as classical monocytes, but we termed them Mo1 in our assay to avoid any confusion; the other three subsets were labelled in a similar way. Roughly 10% of all monocytes express CD16 and were subdivided into the CD14^lo^/CD16^+^ Mo2 subset (also referred to as non-classical monocytes) and the CD14^hi^/CD16^+^ Mo3 subset (often referred to as intermediate monocytes). Following the identification of these three monocyte subsets, a fourth subset remained which expresses CD14 at low levels and no CD16. These cells were labeled as Mo4 despite that these cells may not be monocytes per se, but rather DC precursors [78]. As this subset may have a very low or even no HLA-DR expression, it can also be found as CD14^+^/HLA-DR^−/lo^ monocytes in the literature and has already been associated with cancer prognosis [79].

In order to determine the activation state of the monocytes, the expressions of CD80, CD86, CD64 (FcγRI) and HLA-DR were investigated (Figure 6C–F). There is no or only very weak expression of CD80 on resting monocytes (Figure 6C), which has been described to be upregulated upon stimulation only [80]. In contrast, almost all monocytes constantly expressed CD86 and CD64 which, however, both might be further upregulated upon activation [80,81]. Thus, both markers were analyzed in two patterns (Figure 6D,E). Likewise, the HLA-DR is constantly expressed on most monocytes, but may be upregulated [82] or lost [83] under certain diseased conditions. This regulation or loss of HLA-DR has already been described in several studies as a prognostic marker for severe inflammation with connection to therapy outcome [84,85,86,87,88,89,90,91,92,93,94], but the continuous expression of HLA-DR may complicate the evaluation of its up or downregulation in patient samples. However, as performed for CD64 and CD86, the expression patterns could roughly be divided into two non-separated patterns which were defined and analyzed as HLA-DR^+^ (normal state) and HLA-DR^hi^ (upregulated state) as shown in Figure 6F. In defining gate boarders, the comparison of the four subsets might be useful. With the help of Boolean gates these activation states might easily be further evaluated on the subset level of monocytes (not shown*).*

### 2.8. Granulocytes

The granulocytes comprise the neutrophils, eosinophils and basophils. The first two have very characteristic high SSC properties and are therefore easy to identify; this is further improved by the expression of CD66 as a typical pan marker [95]. In contrast, the basophils show no typical scatter characteristics and are located in-between the lymphoid (SSC^lo^) and monocyte (SSC^int^) populations. They also lack the expression of a characteristic pan marker and therefore are often not examined in patient blood. However, these cells seem to have a regulatory potential [96] and therefore their characterization could be worthwhile.

In the DIoB assay the neutrophils and eosinophils were identified in panel P09 (Table 1: pink row) by their high SSC attributes and joint expression of CD66 (Figure 6G). Then, they were distinguished from each other by their differential CD16 expression [97] (Figure 6H). The neutrophils express, in contrast to NK cells and monocytes, the isoform B of the CD16 molecule and we included the 3G8 clone for its examination as already described above in the NK cell section. Subsequently, the neutrophils and eosinophils were investigated for their expression of CD64, which is, in contrast to the constitutive expression on monocytes, expressed on neutrophils and eosinophils upon stimulation only [98,99] (Figure 6I,J).

The basophils were identified together with DCs in P10 (Table 1: violet row). As both cell types lack characteristic identification markers, the first step in their identification is the exclusion of all other immune cells. Therefore, a lineage cocktail (LIN) was introduced comprising pan markers (CD3, CD14, CD16, CD19, CD20 and CD56) for identification and exclusion of those cells (Figure 6K). Then, within the remaining cells the basophils were identified by their HLA-DR negative and CD123 positive phenotype [100,101] (Figure 6M,L).

### 2.9. Dendritic Cells

The DCs are spread over the whole body predominantly residing the tissues, but also circulating the periphery in an immature form. Even though they circulate in very small numbers counting for less than 1% of all leukocytes, they are potent regulators of the immune system and thus even small modulations could cause extensive effects. It is well described that blood DCs do not express any specific lineage markers, but are positive for HLA-DR and can then further be subdivided by their expression of CD11c, CD123 and CD1c (overview of DC subsets provided in [102,103]).

The DCs were determined together with basophils in P10 (Table 1: violet row) as for both immune cell types the discrimination of all other immune cells is crucial. Therefore, a lineage cocktail (LIN: CD3, CD14, CD16, CD19, CD20 and CD56) was included eliminating the unwanted immune cells (Figure 6K). In the next step, DCs were identified by their expression of HLA-DR (Figure 6L) and then divided into the two major subsets of plasmacytoid (pDC: CD123^hi^/CD11c^−^) and myeloid DCs (mDC: CD11c^hi^/CD123^−^) as shown in Figure 6N. The mDC were further classified by their CD1c expression into type 1 (mDC-1, CD1c^+^) and type 2 (mDC-2, CD1c^−^) [104,105,106] (Figure 6O). The mDC-2 are characterized as CD141^+^, but are negative for CD1c [106,107]. For simplification we gated on the CD1c^−^ fraction. However, one might also add the CD141 antibody as an additional marker. Due to the very low frequency of the mDC-2 subset, we stained 300 µL of whole blood to obtain sufficient cell numbers.

Eventually, the DC subsets were investigated for their expression of the immune tolerance inducing CD274 (PD-L1: Programmed Cell Death Ligand 1) [108,109,110] as shown in Figure 6P. Therefore, the Boolean gate all DCs was defined (see Table 3). This expression was then also compared to the CD279 (PD-1) expression on T cells as these interactions are key immune checkpoint regulators (summarized in [33]). Although it has been described that most circulating DCs in the peripheral blood are immature [111,112], the expression of the common maturation marker CD83 was analyzed (Figure 6P).

### 2.10. Non-Immune Cells

In addition to the manifold immune cell subsets also some non-immune cells were identified which circulate the blood in a very low frequency. These include the hematopoietic stem cells (HSC), endothelial progenitor cells (EPC) and circulating endothelial cells (CEC). In healthy persons these cell types are very rare counting for less than 0.01% of all white blood cells, but were described to become more frequent in various diseases and cancer. In the steady state the HSC predominantly replenish myeloid immune cell subsets in the peripheral tissues, but an increased number was connected to immunosuppression and disease progression in various solid tumors [113]. The EPC and CEC play both important roles in neovascularization and angiogenesis and were also associated with poor therapy outcome in cancer [114,115].

**Figure 7:**
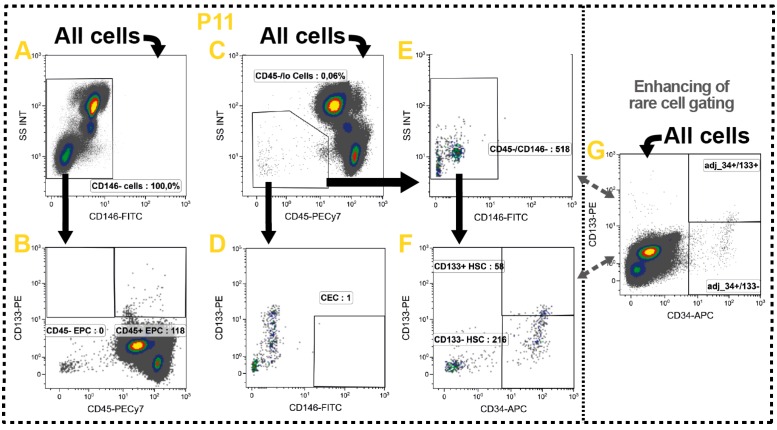
Gating strategy for the identification circulating non-leukocytes which have been associated with cancer therapy outcome. (**A**,**B**) The EPCs were identified by their lack of CD146 while expressing CD133 and directly divided into CD45^+^ and CD45^−^ EPC; (**C**,**D**) The CECs express no or just little CD45 and lack CD133, but are positive for CD146; (**C**,**E**,**F**) Likewise, the HSC express no ore just little CD45, but lack CD146 and are positive for CD34. In the gating process they were directly distinguished into CD133^−^ and 133^+^ HSC; (**G**) As all these cell types are very rare in most persons, the CD34 and CD133 gates were aligned according to its expression on the all cells level; and (**A**–**G**) Black arrows represent the gating strategy and the depicted percentages depend on the respective input gate.

These rare and heterogeneous cell populations are characterized in P11 (Table 1: yellow row) evaluating the expression of CD34, CD146 and CD133 in addition to the common leukocyte marker CD45 [116] (Figure 7). Here, one has to stress that no precise phenotypic definitions exist for identification of these rare cells. However, consensus was found in defining CECs as CD146^+^ and CD133^−^ [115,117,118,119]. In contrast, the EPCs lack CD146, but express CD133 [116,117,120,121]. The HSCs are generally characterized as CD45^−/lo^ and CD34^+^. Furthermore, they normally lack CD146 and potentially express the hematopoietic marker CD133 [117,122].

Based on these definitions, we gated on all CD146^−^ cells which also express CD133 to identify EPCs (Figure 7A,B). As various groups described that there are CD45^−^ and CD45^+^ EPC, we also took this into consideration (Figure 7B). Then, we identified the circulating HSC which express no or only little CD45 and lack CD146, but are positive for CD34 (Figure 7C,E,F). As there are reports about CD133^+^ and CD133^−^ HSC, one might include this distinction additionally (Figure 7F). In parallel, the CEC were identified as cells also expressing no or only little CD45, but in contrast to HSC are CD146^+^/CD133^−^ (Figure 7C,D). Due to the low event number the CD34 and CD133 gates were adjusted on the All cells-level (Figure 7G).

We prepared 300 µL of whole blood for the analyses as these cells are very rare. This volume might be further elevated to also identify those cells in healthy persons. Here, one should consider that due to the low number of cells and lack of consensus in phenotypic characterization, a collective identification might be appropriate. Therefore, one could define these cells as hematopoietic stem and progenitor cells (HSPC) and sum the individually identified cells using Boolean gates. Alternatively, a simplified gating strategy could be applied.

### 2.11. Determination of Absolute Cell Numbers

Since leukocyte numbers vary during therapies and the DIoB assay will especially be used for longitudinal monitoring of patients during therapy, we additionally determined the absolute leukocyte count in panel P12 (Table 1: uncolored row). Therefore, the blood was transferred into a TruCount tube from BD Bioscience containing a definite number of beads and stained with antibodies against various pan markers (CD3, CD16, CD19, CD20, and CD56). Then, cells and beads were simultaneously acquired allowing the determination of the leukocyte count in the blood sample.

**Figure 8:**
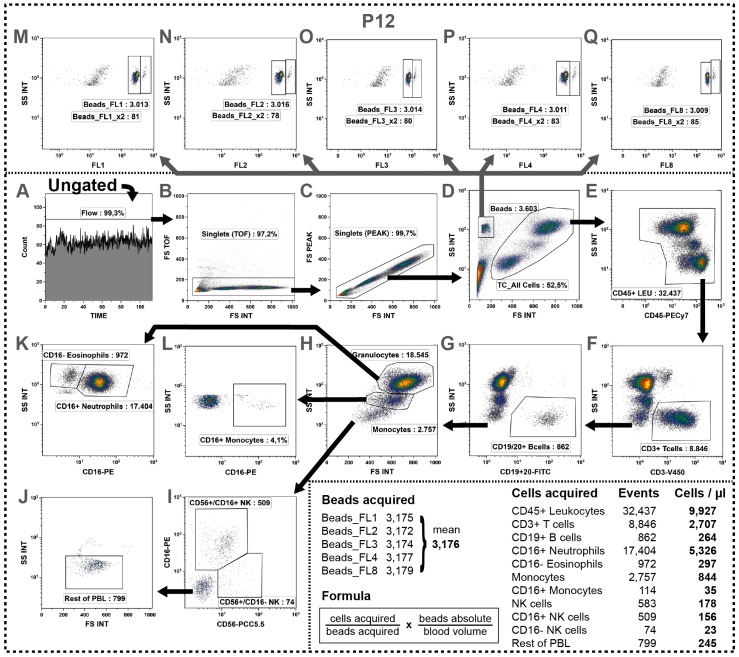
Determination of absolute cell counts by a simultaneous acquisition of cells and beads. (**A**–**D**) Similar to all other panels, first irregularities were excluded from analysis, followed by doublet discrimination and the definition of the All Cells-gate; (**E**–**J**) Then, various major cells were determined keeping a strict gating order always excluding the already identified cell types from the next gating step by the use of Boolean gates (termed as Rest); (**E**) First, leukocytes were discriminated from debris by their CD45 expression; (**F**) Then, the CD3^+^ T cells were identified; (**G**) followed by B cell identification by their expression of CD19 or CD20 within the CD3^−^cells; (**H**) Within the remaining cells the granulocytes and monocytes were detected by their particular scatter characteristics; (**I**) followed by detection of NK cells expressing CD56 and/or CD16; (**Q**) Then, all left-over cells were defined as Rest of cells containing non-determined cells such as DCs, basophils or HSCs; (**K**,**L**) Besides, the granulocytes and monocytes were further subdivided by their CD16 expression into eosinophils and neutrophils or CD16^+^ and CD16^−^ monocytes respectively; (**D**) In parallel, the bead count was determined. Therefore, a Beads-gate was defined based on its dense scatter characteristics whereby it was important not to exclude the small beads together with debris; and (**M**–**Q**). Then, these beads were verified by their auto fluorescence properties. We found two populations in the FL1, FL2, FL3, FL4 (**blue laser**) and FL8 (**red laser**) representing a major singlet and a minor doublet population. Consequently, for each channel we added the doublet population twice to the singlet population and calculated the mean value out of all five channels (see **table at the bottom**). Then, using the indicated formula, the absolute cell count per µL of initial blood was calculated for all acquired cells as shown for the representative example.

In the first step, the morphologic properties were, similar to the gating of the other 11 panels, examined to discriminate any unwanted events. However, here, the gate settings were slightly adapted to the elevated FSC properties as this sample has not been centrifuged. Thus, a Flow-gate, two singlet-gates and the All Cells-gate were defined (Figure 8A–D). As no washing steps were performed, the leukocytes were discriminated from debris defined by their CD45-expression (Figure 8E) which was not necessary in the other panels. In order to identify all major immune cells the gating was performed in the following sequence: T cells (CD3^+^), B cells (CD19^+^ or CD20^+^), monocytes (FSC vs. SSC), granulocytes (FSC vs. SSC), NK cells (CD56 vs. CD16) and Rest of PBL (Figure 8F–J). Thereby, the already identified cells were excluded from the next analysis step resulting in enrichment of the down-stream identified smaller immune cell populations. Additionally, the CD16 expression of granulocytes and monocytes (Figure 8K,L) was examined.

In parallel, the number of acquired beads was determined. Therefore, a Beads-gate (FSC^lo^/SSC^hi^) was created (Figure 8D) and the fluorescence emission of the beads was checked in all fluorescence channels. Using our cytometer settings (see Table S2), the fluorescence channels FL1, FL2, FL3, FL4 (blue laser; Figure 8M–P), and FL8 (red laser; Figure 8Q) were most suitable for a distinct visualization of the beads. The identification of beads was very clear and without any variations between the fluorescence channels. Nevertheless, we observed two populations with the same SCC but different FSC characteristics concluding a singlet (>97%) and a doublet (<3%) population. Thus, the doublets were added twice to the singlets and the mean of all five channels was calculated.

Finally, we obtained the absolute cell number for all determined cell types per µl of blood using the formula stated at the bottom of Figure 8: (“cells acquired”/“beads acquired”) × (“absolute beads per tube”/“absolute blood sample volume”). These absolute cell counts were then transferred onto the major cells determined in the other eleven panels allowing the calculation of an absolute number for all 37 cell subsets determined in the DIoB assay.

### 2.12. Determination of a General Assay Robustness

**Figure 9:**
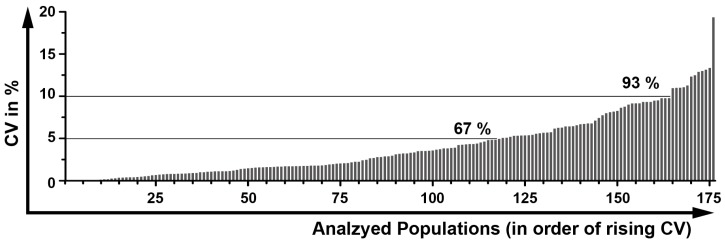
General robustness of the DIoB assay. Three whole blood samples of two different healthy donors were independently processed, measured and analyzed. These were analyzed by calculating the percentage distribution for 208 populations. Thereof, all populations counting for less than 100 events were excluded. In the analysis of these NHD blood samples 176 valid populations remained for determination of variations. Therefore, the coefficients of variation (CVs) were separately calculated for both samples. Then, the mean values were calculated representing the general robustness of the DIoB assay. The graph shows that the majority of populations (67%) had a CV below 5% and nearly all populations (93%) had a CV below 10%. Only 11 populations had a CV between 10% and 15% and one above 15%.

In order to determine the robustness of the DIoB assay, we processed and analyzed the blood samples of two normal healthy donors (NHD) three times separately and calculated the CV between all populations (Figure 9). In total, the proportional distributions of 208 populations were calculated. Hereby, we observed that high CV values only occurred within small populations and within the activation state determinations, as also described by others. Thus, all populations that consisted of less than 100 events were excluded from further interpretation as an informative value cannot be granted.

The evaluation of the remaining 176 populations showed that most populations (67%) had a very small CV below 5% (Figure 9), and nearly all populations (93%) had a small CV below 10%. Only 11 populations (6%) had a CV between 10 and 15% which were all rather small subsets (<0.1% of leukocytes) or low-frequently expressed activation markers. In the end, only one population (CD80^+^ monocytes) had with 19.8% a CV above 15% which is an activation marker almost not expressed on resting cells in blood samples of NHD.

## 3. Discussion

Today, the immune monitoring not only focuses on the distinction of particular cell subsets, but also on the interactions between these cells. Furthermore, as so far no single biomarkers have been identified that are connected to cancer progression or therapy outcome, one searches for groups of markers that jointly reveal their predictive or prognostic potential [123]. Consequently, the here presented DIoB assay was designed for recognizing a multitude of immune cells and its subtypes that circulate the blood including granulocytes.

In the past, neutrophils and eosinophils were often overlooked, but recently both cell types became increasingly important as they might carry regulatory potential in various diseases [124,125,126], which also was described for basophils [96]. Their identification has been feasible, as the DIoB assay was established for a direct staining of whole blood samples. Many immune monitoring assays require the isolation of PBMC prior to the staining procedure [18,19,127]. In contrast to whole blood samples, this isolation of cells allows long-term shipments and even sample storage by cryopreservation. However, one has to keep in mind when designing translational monitoring assays to adapt them to the respective needs of study. PBMC isolation procedures exclude neutrophils and eosinophils from investigation and cryopreservation might strongly impact on DC [18]. Moreover, the sample preparation is more complex compared to a whole blood staining, resulting in increased expenditure of time, working capacities and potential sources of errors. Furthermore, direct staining of whole blood samples reduces the loss of cells and minimizes the alteration of the cell phenotype. It already has been reported that the isolation [128] or the freezing/thawing-cycles of isolated PBMC [129] can alter the expression of certain surface markers. And most important, the direct staining procedure allows the determination of an absolute cell count as one should always consider that leukocyte numbers might vary during therapy. Thus, in- or decreasing leukocyte numbers can be followed that would remain hidden in proportional analysis. Likewise, the in- or decrease of one subset might alter the percentages of other subsets in proportional analysis even though these remained unchanged in total numbers.

The DIoB assay is a robust multicolor flow cytometry-based assay in a modular design allowing the identification of up to 37 different cells circulating in the peripheral blood. These include all major immune cell types and three additional non-immune cell subsets which have already been associated with therapy outcome. In addition, several activation markers are determined to evaluate the phenotype of the identified cells. The assay robustness is high as increased CV values only occurred in the very small populations. Consequently, all gates containing less than 100 events were excluded from interpretation as previously described and suggested [14,130]. Even though studies on minimal residual diseases have shown that cells can also be identified by less than 100 events [131], we excluded such populations from interpretation as the focus was not set on such very rare cells. Nevertheless, we elevated the amount of blood in the panels P10 and P11 to allow the detection of some low-frequency cells.

One has to stress that also some disadvantages arise with the DIoB assay: the antibody costs per measurement are not low. Additionally, the costs for cytometer and staff have to be taken into account. Furthermore, the first establishment of the assay requires experiences and consumes time. Moreover, the analysis procedures need to be coordinated, as huge amounts of data have to be analyzed.

Recently, Finak et al. [19] reported that in multi-centric studies the intra-site variability is generally low, but the inter-site variability is a major problem, which also was confirmed by others [18,20,127]. This predominantly was due to unintended deviations in preparation and analyses between the different sites even though detailed SOPs (standard operating procedures) were provided [19]. Such variations due to preparation purposes can be reduced by a centralized training of the technical staff as described by Streitz et al. [20]. However, the most prominent variations were due to subjectivity during analyses and thus it is general consensus to perform centralized [18,19,20,127] or automated analyses [19,132]. The flow cytometry community and future clinical trials greatly benefit from such studies for harmonization of multi-centric trials. Likewise, single-center trials profit from it, but here the establishment of flow cytometry assays has wider margins. The DIoB assay was developed for trials which go along with a centralized preparation of fresh blood samples. Consequently, the inter-site variability does not apply and also the on-site variability has been no problem as shown by the low CV values. Thus, the DIoB assay is well-suited for clinical trials or routine examinations which allow a centralized sample preparation and analysis. Nevertheless, as it was optimized for an easy, fast and consistent sample preparation with a limited number of steps, an application of the DIoB assay in multi-centric trials is also feasible. The requirement of a maximum of eight different fluorescence channels makes this assay suitable for all modern cytometers. And the repeated use of antibodies in different panels reduces the effort in establishing this assay to suit the local requirements. We measured all panels using the same cytometer settings (see Table S2) which strongly simplifies the transfer of this assay. Nevertheless, attention should be paid during analysis as the different antibody combinations required individual compensation values leading to varying signals in both, the negative and the positive populations. This, however, was balanced by adjusting the logicle scales independently for every parameter of each panel.

In conclusion, the DIoB assay is a robust multicolor flow cytometry assay for identification of circulating immune cell subsets, as well as non-immune cells and additional activation markers in whole blood samples. The modular design makes the DIoB assay suitable for a wide range of applications as one may select, depending on the study objectives, the desired panels which each are dedicated to characterize a certain cell type. This might be important in explorative studies as one can assess a comprehensive immune status at the beginning and reduce it later on by still allowing direct comparisons. Moreover, it is relatively simple to adapt existing panels by adding or replacing certain activation or subset markers. Likewise, the blood withdrawal of only 2.0 mL is a small burden for most patients. Further, the direct staining of cell surface proteins in whole blood samples also decreases preparation effort, time, and variations. Worthy of note is that the assay is suited for the measurement on every cytometer that is capable of determining at least 8 different colors. Merely, the fluorochromes or filter settings need to be adapted to suit the local requirements. Everything else, like sample preparation, antigens, antibody clones or gating strategy could be applied directly. As a consequence, one could assess therapy effects of single patients and thus estimate individual responses. This contributes to the further development of personalized therapies and to the identification of immune biomarkers. The DIoB assay has already been applied for clinical immune monitoring of cancer patients. Analyses of patients with glioblastoma multiforme (IMMO-GLIO 01 trial, NCT02022384), pancreatic cancer (CONKO-007 trial, NCT01827553), and head and neck cancer (DIREKHT trial, NCT02528955) are currently ongoing.

## 4. Materials and Methods

### 4.1. Blood Withdrawals from Healthy Donors

For the establishment of the DIoB assay, the peripheral blood was drawn from 15 healthy volunteers using EDTA-Monovette tubes (Sarstedt, Nümbrecht, Germany) and processed within 4 h. This was approved by the ethics committee of the Bayerische Landesärztekammer (#12131) in accordance with the principles described in the current version of the Declaration of Helsinki. All donors accepted and provided written informed consent.

### 4.2. Choice of the Antibodies and Preliminaries

Based on literature review and previous experiences, we focused on 37 different immune and non-immune cell subsets for investigation. For their distinct identification, the required surface antigens and additional activation markers were assigned. Subsequently, antibodies were selected according to vendor availability and with regard to common practice for choosing fluorochromes, such as using bright fluorochromes for low expressed antigens and the other way around. We also took into consideration that antibody clones recognizing different epitopes could lead to varying signals between different cell types. The panel overview is provided in Table 1 and further details on applied antibodies are available in Table S1. All antibodies were titrated in whole blood samples and cross-checked for specificity against their unstained and their respective isotype controls. The titrations were estimated for signal to noise ratio and only distinct positive signals were accepted. Then, best antibody dilutions were combined to the panels and cytometer settings were revised.

In order to determine the compensation values, we stained VersaComp Antibody Capture Beads (Beckman Coulter, Krefeld, Germany) according to the manufacturer’s instructions and calculated them using the Kaluza analysis software (version 1.3, Beckman Coulter). These compensation values were then cross-checked in single-staining of whole blood samples in comparison to their according fluorescence minus one (FMO) controls which make false positive events clearly visible. The FMO controls were moreover beneficial for the exact discrimination between positive and negative populations allowing the definition of gate settings for later analyses. The antibody concentrations (Table S1), cytometer settings (Table S2) and compensation values were then transferred to the final panels.

### 4.3. Sample Preparation

The samples were always handled at room temperature until fixation and kept on ice afterwards. Cooling of the blood prior to fixation can lead to degranulation of the neutrophils which might cause further damage to other cells, thereby altering the sample condition. However, following fixation they should be stored refrigerated if an immediate measurement is not possible. The measurement should be performed within 4 h. In order to standardize sample preparation, we defined SOPs allowing an easy procedure of the DIoB assay with low variations. It can be handled by any person who works according to these SOPs.

Prior to the staining procedure an antibody master mix was prepared for every panel based on the previous titrations (Table S3). Then, for all panels, except P12, 100 µL of whole blood were distributed into 5 mL polypropylene tubes (Sarstedt) and stained with the respective antibody mix for 25 min at room temperature in the dark. To obtain sufficient cell numbers within the panels P10 and P11, three times 100 µL of blood were stained in parallel and pooled directly before measurement. Simultaneously, the TruCount tubes (BD Biosciences, Heidelberg, Germany) containing counting-beads for the determination of absolute leukocyte numbers in P12 were prepared according to the manufacturer’s instructions. In short, dry beads were resolved in 20 μL of antibody mix and 50 μL of whole blood were added. Then, together with the other tubes they were incubated for 25 min at room temperature in the dark.

In the next step, the stained blood samples were prepared in a standardized manner with an automated 3-step process using the TQ-Prep Workstation (Beckman Coulter) according to the manufacturer’s instructions. In short, first erythrocytes were lysed with formic acid, then leukocytes were re-buffered with carbonate and finally, a fixation with paraformaldehyde (PFA, Sigma-Aldrich, Taufkirchen, Germany) was performed. This TQ-prep-Workstation from Beckman coulter provides an automated sample preparation under constant conditions. Obviously one could also manually lyse and fixate the blood. Therefore, first 600 μL of formic acid (0.12% in ddH_2_O) are added to the stained blood sample and gently vortexed for 10 s. Then, 265 μL of carbonate buffer (56.6 mM Na_2_CO_3_ (Merck-Millipore, Darmstadt, Germany), 248.1 mM NaCl (Carl Roth, Karlsruhe, Germany) and 219.0 mM Na_2_SO_4_ (Sigma-Aldrich) in ddH_2_O) are added and immediately vortexed for 10 s. Finally, 100 µL of phosphate-buffered saline (PBS, Sigma-Aldrich) containing 1% of PFA are added and also vortexed for 10 s (for preparation of solutions see Table S4).

Finally, all samples, except P12, were washed twice with PBS (300× *g*, 5 min) and pellets were dissolved in 200 µL of PBS containing 1% of PFA. As far as possible, the samples were measured immediately. Otherwise, they were kept on 4 °C in the dark for a maximum of 4 h until acquisition.

### 4.4. Data Acquisition

The samples were measured on the Gallios flow cytometer (3 Laser, 10 colors, standard filter configuration) from Beckman Coulter. The cytometer settings (photomultiplier tubes, compensation) were based on the optimizations described above (see Table S2). For all acquisition protocols, the same scatter settings were defined and fluorescence channels not in use were deactivated. The number of total events required for a clear discrimination of the subsets was defined for each panel and adjusted through measurement duration or indirectly through the acquired sample volume. Thus, for panels which were focused on rare subsets or deep sub gating the complete sample amount of 200 µL (equivalent to 100 µL of initial blood sample) was acquired, but for panels focusing on more frequent subsets only 50%–70% of the sample volume was needed. For the panels P10 and P11 the complete sample volume of 600 µL (equivalent to 300 µL of initial blood) was used.

### 4.5. Data Analysis

The obtained data were analyzed using the Kaluza analysis software (version 1.3). Therefore, a composite protocol with the ability to directly link multiple acquisition files and thereby connecting the different data files of the same sample was created. This allowed an easy definition of the same gates for multiple files which reduced variations. If the local analysis software does not support such a linking feature, one of course could also transfer the gate settings from one file to the other still guaranteeing the analysis of the same populations. To define gate boarders, positive and negative populations were compared to FMOs, single and isotype stainings. Based on these definitions, the gates were adjusted for varying signals due to different cell numbers during the analyses of patient samples.

All parameters were analyzed using density dot plots. The FSC signals were examined in linear scales and the SSC signals were recorded logarithmically. In contrast, all fluorescence parameters were analyzed using logicle (bi-exponential) scales (as proposed by [133,134]) and their scale settings (decades, negative percentages) were adjusted separately for each parameter, but kept constant between all analyses. This resulted in a better visualization and distinction of positive, dim, and negative populations as lower signals no longer stuck to one axis. In addition, over or undercompensated data can be displayed very clearly [133]. Thus, it was possible to detect collapsing fluorochrome dimers by cross-checking compensations in every analysis preventing false results.

Finally, the raw data were exported into MS Excel (Microsoft, Redmond, USA) to calculate the proportional distribution of all determined subsets. First, we calculated the percentages of every major cell type (e.g., all CD3^+^ T cells) out of all cells. Then, every subset was calculated out of its major cell type (e.g., CD4^+^/CD8^−^ T_H_ of all T cells). The activation states were determined by calculating all positive events in relation to their input gate. Finally, we transferred the absolute cell numbers determined for all leukocytes and the main cell types in P12 onto the other panels to determine the absolute cell number per milliliter of blood for each investigated subset.

### 4.6. Assay Robustness

The assay robustness was determined by the individual processing and analyzing of blood samples from two volunteers for three times. We then calculated the mean and the coefficient of variation (CV in %: standard deviation/mean × 100) for all 208 obtained populations.

## Figures and Tables

**Table 1:** Overview of the 12 staining panels each dedicated to a specific cell type which is indicated by individual colors. Laser and filter settings as well as the applied fluorochromes which are coupled to the monoclonal antibodies that are directed against the antigens listed beneath are depicted. The panels examine T cells (**red**: P01–P03, P05h), B cells (**green**: P04, P05), NK cells (**blue**: P06, P07), monocytes (**brown**: P08), neutrophils and eosinophils (**pink**: P09), basophils and dendritic cells (**violet**, P10) as well as circulating non-leukocytes (**yellow**: P11). The last panel (P12) contains counting beads and antibodies directed against several major cells to determine the absolute cell count for all identified subsets.

Panel	Blue: 488 nm				Red: 638 nm		Violet: 405 nm	
	525/40	575/30	695/30	755/LP	660/20	755/LP	450/50	550/40
	FITC|A488	PE	PCC5.5	PECy7|PEV770	APC	APCH7	V450|BV421	KO
**P01**	CD8	CD197	CD4	CD45RA	CD38		CD3	
**P02**	CD127	CD196	CD4	CD25	CD183		CD3	
**P03**	TCRγ/δ	TCRα/β			CD152		CD3	
**P04**	CD20		CD24	CD5	CD38		CD27	CD19
**P05**	CD19/20	CD25	CD86	CD69	CD279	CD80	CD3	HLA-DR
**P06**	CD69	CD16	CD56		CD314		CD25	CD3
**P07**	CD159c	CD16	CD56	CD94	CD159a			CD3
**P08**	CD14	CD16	CD86		CD80		CD64	HLA-DR
**P09**	CD66						CD64	CD16
**P10**	LIN ^1^	CD274	CD123	CD83	CD1c		CD11c	HLA-DR
**P11**	CD146	CD133.1		CD45	CD34			
**P12**	CD19/20	CD16	CD56	CD45			CD3	

^1^ LIN includes CD3, CD14, CD16, CD19, CD20 and CD56.

**Table 2:** Definition of monitored cell subsets.

Cell Subset			Definition
Leukocytes			Forward scatter (FSC) vs. Side scatter (SSC)
T cells			CD3^+^
T helper cells (T_H_)			CD3^+^/CD4^+^/CD8^−^
	T_H_1		CD3^+^/CD4^+^/CD8^−^/CD183^+^/CD196^−^
	T_H_2		CD3^+^/CD4^+^/CD8^−^/CD183^−^/CD196^−^
	T_H_17		CD3^+^/CD4^+^/CD8^−^/CD183^−^/CD196^+^
	T_REG_		CD3^+^/CD4^+^/CD8^−^/CD25^hi^/CD127^−/lo^
	Naïve T_H_		CD3^+^/CD4^+^/CD8^−^/CD197^+^/CD45RA^+^
	Effector T_H_		CD3^+^/CD4^+^/CD8^−^/CD197^−^/CD45RA^+^
	EM T_H_		CD3^+^/CD4^+^/CD8^−^/CD197^−^/CD45RA^−^
	CM T_H_		CD3^+^/CD4^+^/CD8^−^/CD197^+^/CD45RA^−^
	Cytotoxic T cells (T_C_)		CD3^+^/CD8^+^/CD4^−^
	Naïve T_C_		CD3^+^/CD8^+^/CD4^−^/CD197^+^/CD45RA^+^
	Effector T_C_		CD3^+^/CD8^+^/CD4^−^/CD197^−^/CD45RA^+^
	EM T_C_		CD3^+^/CD8^+^/CD4^−^/CD197^−^/CD45RA^−^
	CM T_C_		CD3^+^/CD8^+^/CD4^−^/CD197^+^/CD45RA^−^
	TCRα/β T cells		CD3^+^/TCRαβ^+^/TCRγδ^−^
	TCRγ/δ T cells		CD3^+^/TCRγδ^+^/TCRαβ^−^
B cells			CD19^+^ or CD20^+^
	Pre^−^naïve B		CD19^+^ or CD20^+^/CD27^−^/CD38^−/lo^/CD5^+^
	Naïve B		CD19^+^ or CD20^+^/CD27^−^/CD38^−/lo^/CD5^−^
	Memory B		CD19^+^ or CD20^+^/CD27^+^/CD38^−/lo^/CD5^−^/CD24^+^
	Transitional B		CD19^+^ or CD20^+^/CD27^−^/CD38^hi^/CD5^+^/CD24^hi^
	Plasma blasts		CD19^+^ or CD20^+^/CD27^+^/CD38^hi^/CD5^−^/CD24^−^
	B_REG_		CD19^+^ or CD20^+^/CD27^+^/CD24^hi^ following the exclusion of the other five B cell subsets (see Table 3: Rest of B cells)
NK cells			CD3^−^/CD56^+^
	NK1		CD3^−^/CD56^+^/CD16^+^
	NK2		CD3^−^/CD56^hi^/CD16^−^
	NK3		CD3^−^/CD56^lo^/CD16^−^
NKT			CD3^+^/CD56^+^ or CD3^+^/CD16^+^ or CD3^+^/NKG2D^+^
Neutrophils			CD66^+^/CD16^+^
Eosinophils			CD66^+^/CD16^−^
Basophils			CD3^−^/CD14^−^/CD16^−^/CD19^−^/CD20^−^/CD56^−^/HLADR^−^/CD123^+^
Dendritic cells			(mDC or pDC)
	mDC		CD3^−^/CD14^−^/CD16^−^/CD19^−^/CD20^−^/CD56^−^/HLADR^+^/CD11^chi^/CD123^−/lo^
		mDC^−^1	CD3^−^/CD14^−^/CD16^−^/CD19^−^/CD20^−^/CD56^−^/HLADR^+^/CD11^chi^/CD123^−/lo^/CD1c^+^
		mDC^−^2	CD3^−^/CD14^−^/CD16^−^/CD19^−^/CD20^−^/CD56^−^/HLADR^+^/CD11^chi^/CD123^−/lo^/CD1c^−^
	pDC		CD3^−^/CD14^−^/CD16^−^/CD19^−^/CD20^−^/CD56^−^/HLADR^−^/CD123^hi^/CD11c^−^
Monocytes			CD14^+^
	Mo1		CD14^hi^/CD16^−^
	Mo2		CD14^lo^/CD16^+^
	Mo3		CD14^hi^/CD16^+^
	Mo4		CD14^lo^/CD16^−^
HSC			CD45^−^/CD146^−^/CD34^+^
CEC			CD45^−^/CD146^+^
EPC			CD146^−^/CD133^+^

**Table 3:** Definition of Boolean gates required for the subset identification.

Panel	Boolean Gate	Definition
P01	TC	“T8^hi^” or “T8^lo^”
P04	CD19 or CD20 B cells	“CD19^+^ B cells” or “CD20^+^ B cells”
P05	Rest of B cells	“CD19 or CD20 B cells” and (not (pre^−^naïve or (naïve or (“memory” or (“transitional” or “plasma blasts”)))))
P06	NKT	“CD3^+^” and (“CD94^+^ NKT” or (“CD56^+^ NKT” or (“NKG2C^+^ NKT” or (“NKG2A^+^ NKT” or “CD16^+^ NKT”))))
P17	NKT	“CD3^+^” and (“CD56^+^ NKT” or (“CD16^+^ NKT” or “NKG2D^+^ NKT”))
P10	All DCs	“MDC” or “PDC”
P01	Rest 1	“CD45^+^ Leu” and (not “CD3^+^ T cells”)
P01	Rest 2	“Rest 1” and (not (“CD19/20^+^ B cells”)
P01	Rest 3	“Rest 2” and (not (“Monocytes” or “Granulocytes”)
P01	Rest 4	“Rest 3” and (not (“CD56^+^/CD16^+^ NK” or “CD56^+^/CD16^−^ NK”)

## Supplementary Materials

Supplementary materials can be found at www.mdpi.com/1422-0067/17/8/1316/s1.

## Abbreviations

### Abbreviations

The following abbreviations are used in this manuscript:

B_REG_	B10 regulatory B cell
CD	Cluster of differentiation
CEC	Circulating endothelial cell
CTLA4	Cytotoxic T lymphocyte-associated protein 4 (CD152)
CV	Coefficient of variation
DC	Dendritic cell
DIoB	Detailed Immunophenotyping of Blood
DNT	Double negative T cell
DPT	Double positive T cell
EPC	Endothelial progenitor cells
FMO	Fluorescence minus one
FSC	Forward scatter
HPC	Hematopoietic stem cell
NHD	Normal healthy donor
P	Panel
PD-1	Programmed cell death protein-1 (CD274)
PD-L1	Programmed death-ligand 1 (CD279)
PFA	Paraformaldehyde
SSC	Side scatter
T_C_	Cytotoxic T cell
T_CM_	Central memory T cell
T_Eff_	Effector T cell
T_EM_	Effector memory T cell
T_H_	T helper cell
T_REG_	Regulatory T cell

### Abbreviations

The following abbreviations for fluorochromes are used in this manuscript:

A488	Alexa-488
APC	Allophycocyanin
APCH7	APC-H7
APCR700	APC-R700
APCV770	APC-Vio770
BV421	Brilliant Violet 421
FITC	Fluorescein isothiocyanate
KO	Krome Orange
PCC5.5	PerCp-Cy5.5
PE	Phycoerythrin
PECy7	PE-Cy7
PEV770	PE-Vio770
V450	Horizon V450
