# Supplementary material for: Development of a Modular Assay for Detailed Immunophenotyping of Peripheral Human Whole Blood Samples by Multicolor Flow Cytometry
Source: Int J Mol Sci. 2016 Aug 11;17(8):1316. doi: 10.3390/ijms17081316 (PMC5000713; doi:10.3390/ijms17081316)
Supplement: Supplementary file 1 [file ijms-17-01316-s001.pdf]

Supplementary Materials: Development of a Modular Assay for Detailed Immunophenotyping of Peripheral Human Whole Blood Samples by Multicolor Flow Cytometry

Paul F. Rühle, Rainer Fietkau, Udo S. Gaipl and Benjamin Frey

Table S1. Details on applied antibodies.

Specificity	Clone	Fluorochrome	Amount per 100 µL Blood	Vendor
CD1c	L161	APC	1.0	eBioscience
CD3	UCHT1	KO	3.0	Beckman Coulter
CD3	UCHT1	V450	1.0	BD Biosciences
CD3	UCHT1	FITC	3.0	BD Biosciences
CD4	RPA-T4	PCC5.5	1.0	BD Biosciences
CD5	UCHT2	PECy7	0.5	eBioscience
CD8	HIT8a	FITC	4.0	BD Biosciences
CD11c	B-ly6	V450	1.0	BD Biosciences
CD14	M5E2	FITC	4.0	BD Biosciences
CD16	B73.1	PE	2.5	BD Biosciences
CD16	B73.1	FITC	2.5	BD Biosciences
CD16	3G8	KO	2.0	Beckman Coulter
CD19	J3-119	KO	1.0	Beckman Coulter
CD19	HIB19	FITC	2.0	BD Biosciences
CD20	2H7	FITC	4.0	BD Biosciences
CD24	ML5	PCC5.5	2.5	BD Biosciences
CD25	M-A251	BV421	2.5	BD Biosciences
CD25	M-A251	PECy7	5.0	BD Biosciences
CD25	M-A251	PE	4.0	BD Biosciences
CD27	M-T271	V450	1.0	BD Biosciences
CD34	563	APC	5.0	BD Biosciences
CD38	HIT2	APC	5.0	BD Biosciences
CD45	HI30	PECy7	1.0	BD Biosciences
CD45RA	HI100	PECy7	2.0	BD Biosciences
CD56	B159	FITC	1.0	BD Biosciences
CD56	B159	PCC5.5	4.0	BD Biosciences
CD64	10.1	V450	2.5	BD Biosciences
CD66	B1.1/CD66 *	FITC	2.0	BD Biosciences
CD69	FN50	PEV770	2.0	Miltenyi Biotec
CD69	FN50	FITC	2.0	Miltenyi Biotec
CD80	2D10	APC	2.0	Miltenyi Biotec
CD80	L307.4	APCH7	5.0	BD Biosciences
CD83	HB15e	PECy7	2.5	eBioscience
CD86	IT2.2	PCC5.5	5.0	BioLegend
CD94	REA113	PEV770	2.0	Miltenyi Biotec
CD123	7G3	PCC5.5	7.0	BD Biosciences
CD127	HIL-7R-M21	FITC	20.0	BD Biosciences
CD133/1	AC133	PE	5.0	Miltenyi Biotec
CD146	P1H12	FITC	2.5	BD Biosciences
CD152	BNI3	APC	10.0	BD Biosciences

Table S1. *Cont.*

Specificity	Clone	Fluorochrome	Amount per 100 µL Blood	Vendor
CD159a	131411	APC	10.0	R&D Systems
CD159c	134591	A488	10.0	R&D Systems
CD183	1C6/CXCR3	APC	3.0	BD Biosciences
CD196	11A9	PE	4.0	BD Biosciences
CD197	150503	PE	5.0	BD Biosciences
CD274	MIH1	PE	20.0	BD Biosciences
CD279	MIH4	APC	10.0	BD Biosciences
CD314	149810	APC	2.0	R&D Systems
HLA-DR	Immu-357	KO	2.0	Beckman Coulter
TCRab	T10B9.1A-31	PE	20.0	BD Biosciences
TCRgd	11F2	FITC	10.0	BD Biosciences

* Instead of the B1.1/CD66 clone one might apply the G10F5 clone which specifically binds to CD66b isotype that exclusively is expressed on neutrophils and eosinophils.

Table S2. Gallios flow cytometer settings.

Laser	Power	Detector	Filter	Range	Voltage	Gain
488 nm	22 mW	FSC	–	–	300	5.0
		SSC	–	–	300	2.0
		FL1	525 BP 40	505–545	470	1
		FL2	575 BP 30	560–590	500	1
		FL3	620 BP 30	605–635	430	1
		FL4	695 BP 30	680–710	630	1
		FL5	755 LP	>755	600	1
638 nm	25 mW	FL6	660 BP 20	650–670	650	1
		FL7	725 BP 20	715–735	–	–
		FL8	755 LP	>755	600	1
405 nm	40 mW	FL9	450 BP 50	425–475	470	1
		FL10	550 BP 40	530–570	400	1

Table S3. Preparation of antibody mix for all panels.

Antigen	Fluorochr.	µL	Antigen	Fluorochr.	µL	Antigen	Fluorochr.	µL
P01: T cells			P05: T & B cell activation			P09: Granulocytes		
PBS	–	22.0	PBS	–	5.0	PBS	–	33.5
CD38	APC	5.0	CD279	APC	10.0	CD64	V450	2.5
CD197	PE	5.0	CD80	APCH7	5.0	CD16	KO	2.0
CD8	FITC	4.0	CD86	PCC5.5	5.0	CD66	FITC	2.0
CD45RA	PECy7	2.0	CD25	PE	4.0	P10: HSC, CEC & EPC		
CD3	V450	1.0	CD20	FITC	4.0	PBS	–	16.5
CD4	PCC5.5	1.0	CD19	FITC	2.0	PI	PI	10.0
P02: TH & TREGs			HLA-DR	KO	2.0	CD133/1	PE	5.0
PBS	–	6.0	CD69	PEV770	2.0	CD34	APC	5.0
CD127	FITC	20.0	CD3	V450	1.0	CD146	FITC	2.5
CD25	PECy7	5.0	P06: NK cells			CD45	PECy7	1.0
CD196	PE	4.0	PBS	–	24.0	P11: DCs & basophils		
CD183	APC	3.0	CD56	PCC5.5	4.0	CD274	PE	20.0
CD3	V450	1.0	CD3	KO	3.0	PI	PI	10.0
CD4	PCC5.5	1.0	CD16	PE	2.5	CD123	PCC5.5	7.0
P03: TCR types			CD25	BV421	2.5	CD14	FITC	4.0
PBS	–	–	CD314	APC	2.0	CD20	FITC	4.0
TCRα/β	PE	20.0	CD69	FITC	2.0	CD3	FITC	3.0
TCRγ/δ	FITC	10.0	P07: NK cells II			CD16	FITC	2.5
CD152	APC	10.0	PBS	–	13.5	CD83	PECy7	2.5
CD3	V450	1.0	CD159a	APC	10.0	CD19	FITC	2.0
P04: B cells			CD159c	A488	5.0	HLA-DR	KO	2.0
PBS	–	24.0	CD56	PCC5.5	4.0	CD56	FITC	1.0
CD38	APC	5.0	CD3	KO	3.0	CD1c	APC	1.0
CD20	FITC	4.0	CD16	PE	2.5	CD11c	V450	1.0
CD24	PCC5.5	2.5	CD94	PEV770	2.0	P12: Absolute cell count		
CD1d	PE	2.0	P08: Monocytes			PBS	–	7.5
CD27	V450	1.0	PBS	–	16.0	CD56	PCC5.5	4.0
CD19	KO	1.0	CD14	FITC	10.0	CD20	FITC	4.0
CD5	PECy7	0.5	CD86	PCC5.5	5.0	CD16	PE	2.5
			CD16	PE	2.5	CD19	FITC	2.0
			CD64	V450	2.5	CD3	V450	1.0
			HLA-DR	KO	2.0	CD45	PECy7	1.0
			CD80	APC	2.0			

All depicted antibody amounts are related to the staining of 100 µL of whole blood.

Table S4. Solutions required for the lysis of erythrocytes and fixation of leukocytes.

**Solution TQ-A: Red Blood Cell Lysis**
0.12% formic acid in ddH_2O
**Solution TQ-B: Leukocyte Re-Buffering**
6.0 g/L sodium carbonate (Na_2CO_3)
14.5 g/L sodium chloride (NaCl)
31.1 g/L sodium sulfate (Na_2SO_4)
Fill up with ddH_2O
**Solution TQ-C: Leukocyte Fixation**
1% Paraformaldehyde in PBS (pH 7.4)
